# Assessing what is needed to resolve a molecular phylogeny: simulations and empirical data from emydid turtles

**DOI:** 10.1186/1471-2148-9-56

**Published:** 2009-03-12

**Authors:** Phillip Q Spinks, Robert C Thomson, Geoff A Lovely, H Bradley Shaffer

**Affiliations:** 1 Department of Evolution and Ecology, Davis, USA; 2 Center for Population Biology, University of California, Davis, USA; 3 Present address Division of Chemistry and Chemical Engineering, California Institute of Technology, Pasadena, USA

## Abstract

**Background:**

Phylogenies often contain both well-supported and poorly supported nodes. Determining how much additional data might be required to eventually recover most or all nodes with high support is an important pragmatic goal, and simulations have been used to examine this question. Most simulations have been based on few empirical loci, and suggest that well supported phylogenies can be determined with a very modest amount of data. Here we report the results of an empirical phylogenetic analysis of all 10 genera and 25 of 48 species of the new world pond turtles (family Emydidae) based on one mitochondrial (1070 base pairs) and seven nuclear loci (5961 base pairs), and a more biologically realistic simulation analysis incorporating variation among gene trees, aimed at determining how much more data might be necessary to recover weakly-supported nodes with strong support.

**Results:**

Our mitochondrial-based phylogeny was well resolved, and congruent with some previous mitochondrial results. For example, all genera, and all species except *Pseudemys concinna*, *P. peninsularis*, and *Terrapene carolina *were monophyletic with strong support from at least one analytical method. The Emydinae was recovered as monophyletic, but the Deirochelyinae was not. Based on nuclear data, all genera were monophyletic with strong support except *Trachemys*, and all species except *Graptemys pseudogeographica*, *P. concinna*, *T. carolina*, and *T. coahuila *were monophyletic, generally with strong support. However, the branches subtending most genera were relatively short, and intergeneric relationships within subfamilies were mostly unsupported.

Our simulations showed that relatively high bootstrap support values (i.e. ≥ 70) for all nodes were reached in all datasets, but an increase in data did not necessarily equate to an increase in support values. However, simulations based on a single empirical locus reached higher overall levels of support with less data than did the simulations that were based on all seven empirical nuclear loci, and symmetric tree distances were much lower for single versus multiple gene simulation analyses.

**Conclusion:**

Our empirical results provide new insights into the phylogenetics of the Emydidae, but the short branches recovered deep in the tree also indicate the need for additional work on this clade to recover all intergeneric relationships with confidence and to delimit species for some problematic groups. Our simulation results suggest that moderate (in the few-to-tens of kb range) amounts of data are necessary to recover most emydid relationships with high support values. They also suggest that previous simulations that do not incorporate among-gene tree topological variance probably underestimate the amount of data needed to recover well supported phylogenies.

## Background

In molecular phylogenetic analysis, it is often the case that some relationships are robust, and relatively "easy" to recover while others are difficult to resolve, leading to phylogenetic hypotheses that consist of a patchwork of well and poorly supported nodes. When difficult nodes are encountered, the next logical step is to add taxa and/or data under the reasonable assumption that additional taxa or characters might enable resolution and/or provide support for poorly supported nodes. Whether it is better to add taxa or data is often dependent on the particular situation. When unresolved nodes are related to a long branch and additional unsampled taxa are available, adding taxa might be preferable to adding characters since including additional taxa can help break up long branches [1-3]. On the other hand, if difficult nodes are encountered among closely related taxa, or if taxon sampling is complete or nearly so, then adding additional characters is probably the better strategy [4]. The amount of data required for resolution of difficult phylogenetic problems associated with short internodes, especially those deep in a tree can represent a particularly difficult challenge [5-7] that often requires massive amounts of sequence data to resolve. However, this is not always the case, and robust species trees can sometimes be recovered from moderate amounts of data. For example, Rokas et al. [8] analyzed 106 genes from eight *Saccharomyces *species and found that data from ≥ 20 genes were sufficient to recover a fully resolved and well-supported species tree, with little additional gain in accuracy as more data were added to the analysis. In general, the amount of data required for a given level of resolution, and the gain in phylogenetic accuracy for an increase in data sampling, depends on the true species tree, the rate of evolution for a particular marker, and the fit of the selected model of evolution to the actual substitution pattern of the data.

Interacting with this general question of data quantity is the more elusive problem of data quality. Individual gene trees may or may not accurately reflect overall phylogenetic trees, rate heterogeneity can lead to long branch attraction [9], and anomalous gene trees can lead to positively misleading phylogenetic results [6]. When combined with the low phylogenetic signal in many nuclear gene sequences, even a few such renegade gene trees can lead to great phylogenetic uncertainty and the need to sample many independent nuclear markers to recover well supported phylogenies. These problems have been further exacerbated in metazoan phylogenetics because of a very heavy reliance on mitochondrial DNA (mtDNA) as a single workhorse molecule; mtDNA is appealing because it is a single-locus genome that often yields very high phylogenetic support, but it is also subject to gene tree-species tree conflicts [5,10] that may require massive amounts of nuclear data to overcome.

### How much data?

Determining how much, and what kind of molecular data will, on average, yield a satisfactory increase in support values for a given phylogeny has been approached in at least two ways. The first is the brute force approach–keep collecting data and track how some measure of precision or accuracy (bootstrap support and among-gene topological concordance are two such measures) does or does not increase. The appeal of this approach is that it conveys a sense of how real data collected on real organisms advances phylogenetic knowledge. However, it has drawbacks: large volumes of sequence data are expensive to collect, and marker development remains a significant technical challenge for many taxa (but see [11-14]). A related approach is to subsample (jackknife) large empirical data sets to determine the minimum amount of data that would have been necessary to recover well-supported trees. In these analyses, "target" phylogenies are generated from large amounts of sequence data, and then subsamples of the full data set are analyzed to determine the fraction of the full data set required to recover the target phylogeny [8,15,16]. Both of these approaches are obviously limited to clades for which large amounts of sequence data are available or can be easily acquired. As a result, the taxa that have been examined in this manner have often been separated by large evolutionary distances and long phylogenetic branches because large sequence resources tend to be distributed widely across clades in a few model organisms.

The alternative strategy, and the one used in this study, is to use a modest multi-gene dataset combined with phylogenetic simulations to explore the predicted gain in support values as more (simulated) data are added to a study. Several previous analyses have used simulations to estimate the amount of data necessary to resolve difficult phylogenetic problems. In some cases, sequence data were "grown" such that one or a few empirical data partitions were bootstrapped to generate progressively larger data sets. These pseudoreplicate data sets were then subjected to phylogenetic analyses to estimate the amount of data potentially required to resolve a phylogeny or recover particular node(s) at a predetermined level of support [4,17-23]. These kinds of approaches also have their strengths and weaknesses. On the positive side, simulated data are essentially free, allowing one to determine ahead of time whether a major sequencing effort might be worth the cost of data acquisition. However, simulated data are never a substitute for the real thing, and the reliability of simulations depends on the models of evolution that are used and idiosyncratic features of the dataset on which the simulations are based. Critically, simulation studies performed to date have not accounted for the effects of topological variation in gene trees on phylogenetic inference, and these effects can be profound. For example, when data from a single locus are pseudoreplicated to create additional simulated characters, even weak signal, when multiplied, can eventually become strong signal, leading to well-supported phylogenies (e.g. [21,23]). Conversely, multigene data sets frequently contain conflicting phylogenetic signal, and analyses performed on these types of data sets will often result in polytomies or recover nodes with poor support. Thus, to realistically simulate data from multiple markers, variation in gene tree topology should be incorporated.

Here, our primary goal is to examine the effect of increasing data on multilocus phylogenetic inference when variation in gene tree topologies is incorporated. We examine this issue using a multi-gene empirical dataset for the new-world pond turtles (family Emydidae) coupled with a simulation approach. Emydids are typical of many real-world clades; some relationships have been well supported since molecular and morphological approaches were first applied to the group, some remain contentious, and our molecular knowledge is based almost entirely on mtDNA (Thomson and Shaffer, in review). Particularly vexing is the frequent conflict among data partitions/analytical methods such that analyses based on morphology or mitochondrial DNA (mtDNA), or employing different methodologies (i.e. maximum parsimony (MP) vs maximum likelihood (ML)) are incongruent. These conflicts are best seen in a recent attempt to summarize previous hypotheses of emydid relationships with a supertree approach. A matrix representation with parsimony (MRP) supertree analysis utilizing all available phylogenies resulted in a tree that was nearly completely unresolved [24].

### Simulation approach

Our goals in this paper are two-fold. First, we present a multi-gene phylogeny of emydid turtles to bring greater resolution to this important vertebrate clade. Given the impending full-genome sequence of the emydid *Chrysemys picta *, the importance of emydids as model systems in ecological, evolutionary and developmental studies [25-29], and the endangered status of many contained species [30], we view this as an important goal in its own right. Second, we use extensive simulation analyses to examine the expected gain in phylogenetic accuracy and precision that will result from an order of magnitude increase in sequence data. We explicitly model the effects of variation in gene tree topology in our work, and explore both the overall gains in phylogenetic resolution, and the ability to recover particular problematic nodes with high support values with a substantial increase in the quantity of sequence data. We did not assess the impact of adding additional taxa because our taxon sampling for deeper nodes in the Emydidae is complete; most of the taxa missing from our analysis represent members of closely-related species groups (*Graptemys*, *Pseudemys*, and *Trachemys*) that offer little opportunity to dissect long branches on the emydid tree. However, species boundaries and intraspecific relationships within these deirocheline genera have long been problematic [31-35], thus these groups will undoubtedly require extensive taxon and data sampling from each putative species as well as the use of newer species-delimitation methods [36-38] for complete resolution.

### Emydid biology

The Emydidae is a clade of 48 currently recognized species [39] of freshwater aquatic, semi-terrestrial, and terrestrial turtles distributed across much of the northern hemisphere from central South America and the West Indies to southern Canada. In addition, one species (*Emys orbicularis*) is distributed across Europe, parts of North Africa, and the Middle East [40], and *Emys trinacris*, which was recently removed from *E. orbicularis*, is narrowly distributed on Sicily, and adjacent mainland Italy [41]. Emydid species diversity is highest in the southeastern United States, where about half of this species diversity is found. Emydids occupy a wide diversity of aquatic and terrestrial habitats including relatively cool lakes, ponds, and streams in southern Canada and the northeastern US (*Chrysemys picta*, and *Emys *[= *Emydoidea*] *blandingii*), brackish coastal habitats along the eastern US seaboard (*Malaclemys terrapin*), freshwater streams and rivers in Mediterranean climates of California (*Emys *[= *Actinemys*]*marmorata*), and terrestrial desert grasslands of the southwestern US/northern Mexico (*Terrapene ornata*) [40].

## Results

### Empirical mtDNA Phylogeny

**Figure 1:**
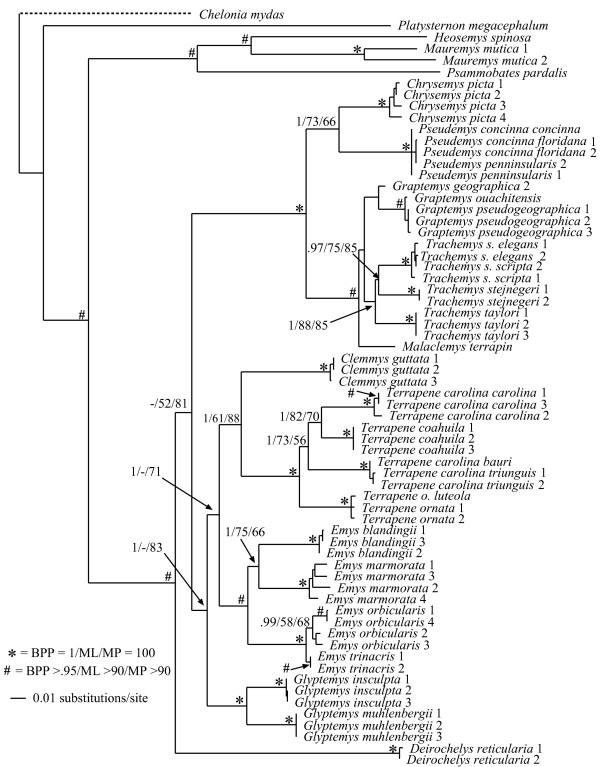
**Maximum-likelihood phylogeny of the 66-taxon mitochondrial cytochrome *b *data set (1070 bp)**. Estimated ML model parameters conform to the GTR+G+I model of sequence evolution. -ln *L *= 8318.58321, rate matrix: A-C = 1.9225, A-G = 16.9646, A-T = 0.9616, C-G = 0.391, C-T = 16.9646, G-T = 1. Base frequencies: A = .30, C = .31, G = .12, T = .27. Proportion of invariant sites (I) = 0.407, and γ-shape parameter = 1.0758. # indicate nodes with Bayesian posterior probabilities (BPP) of 1, and ML and MP bootstrap values of 100. * indicate nodes with ≥ .95 BPP and ML and MP bootstrap values ≥ 90. Numerical values indicate BPP/ML/MP support values.

Visual inspection of the cyt*b *sequencing chromatograms of four samples revealed the presence of multiple peaks at some nucleotide positions, potentially indicating the presence of nuclear mitochondrial pseudogenes (numts) [42]. Since we were unable to confidently determine the actual cyt*b *sequences for these individuals (despite numerous attempts to sequence them), we excluded these sequences from our analysis (Additional file 1). All of the remaining sequences showed the typical mitochondrial composition bias for guanine nucleotides (A = 30%, C = 31%, G = 12%, T = 27%), and the coding region was conserved. Thus, we consider these sequences to represent authentic mtDNA. Our cyt*b *data was composed of up to 1070 base pairs (bp) for 66 taxa. This matrix was mostly complete with ~7% percent missing data. Of the 1070 characters, 567 were constant while 429 were parsimony informative. ML analysis recovered a single tree with a -ln*L *score of 8318.58321. Fig 1 is the ML reconstruction with ML and MP bootstrap values and Bayesian posterior probabilities (BPP) as indicated.

Overall, the mtDNA phylogeny was fairly well supported with most nodes receiving strong support from at least one analytical method (Fig. 1). In addition, all genera and species (for which we had >1 sample) were well supported as monophyletic except *Pseudemys concinna*, *P. peninsularis*, and *Terrapene carolina*. Comparisons of our mitochondrial results to previous analyses were complicated by the fact that phylogenies from previous analyses often vary as a function of analytical method, data partition, and combination of data partitions (see [27]). Thus, depending on the data partition/method(s) used, parts of our mtDNA-based tree were congruent with those from previous analyses while others were novel. For example, the Emydinae was monophyletic, but with support from BPP and MP bootstrap values only (100 and 83, respectively). Relationships among emydine genera recovered here were the same as the ML results, but differed from the MP results, of Feldman and Parham (2002) [43] in their analysis of mitochondrial ND4 and cytochrome *b *gene sequences. Finally, relationships among the Deirochelyinae recovered here were novel, and not congruent with those from previous analyses (e.g. [27,43,44]).

A particularly troubling result from our mtDNA analysis is the placement of *Deirochelys reticularia *as sister to the remaining Emydidae [27], rendering the subfamily Deirochelyinae non-monophyletic. However, *D. reticularia *is on a relatively long branch, thus the phylogenetic position of this taxon might be an artifact due to composition bias of mtDNA sequences. Phylogenetic analyses of R-Y coded data are less susceptible to systematic biases such as composition bias in mtDNA sequences [45] that can lead to spurious phylogenetic results. We used R-Y coding to pool third codon position purines (adenine/guanine: R) and pyrimidines (cytosine/thymine: Y) into two-state categories (R and Y), and performed MP and ML phylogenetic analyses on this data set [45]. Under MP, *Deirochelys *remained sister to the remaining Emydidae. However, under ML *Deirochelys *shifted to a new position within the Emydidae, but the Emydinae was rendered paraphyletic (not shown). Thus, while problematic and in need of final resolution, the relative position of *Deirochelys *based on mtDNA does not appear to be an artifact of composition bias.

### Empirical single-locus nuclear phylogenies

PCR or sequencing reactions failed for seven sequences (despite multiple attempts) so these sequences were coded as missing data (Additional file 1). Patterns from the sequencing chromatograms from all nuclear loci except RAG-1 indicated that some individuals were heterozygous for length polymorphisms [46]. However, by sequencing each gene fragment in both directions, we were able to generate sequence data from most of each locus for the length-polymorphic individuals. In addition, the TB73 locus contained a poly A/T region that was difficult to align confidently so we excluded a 13-bp region of this locus from these phylogenetic analyses [TreeBase S2303].

**Figure 2:**
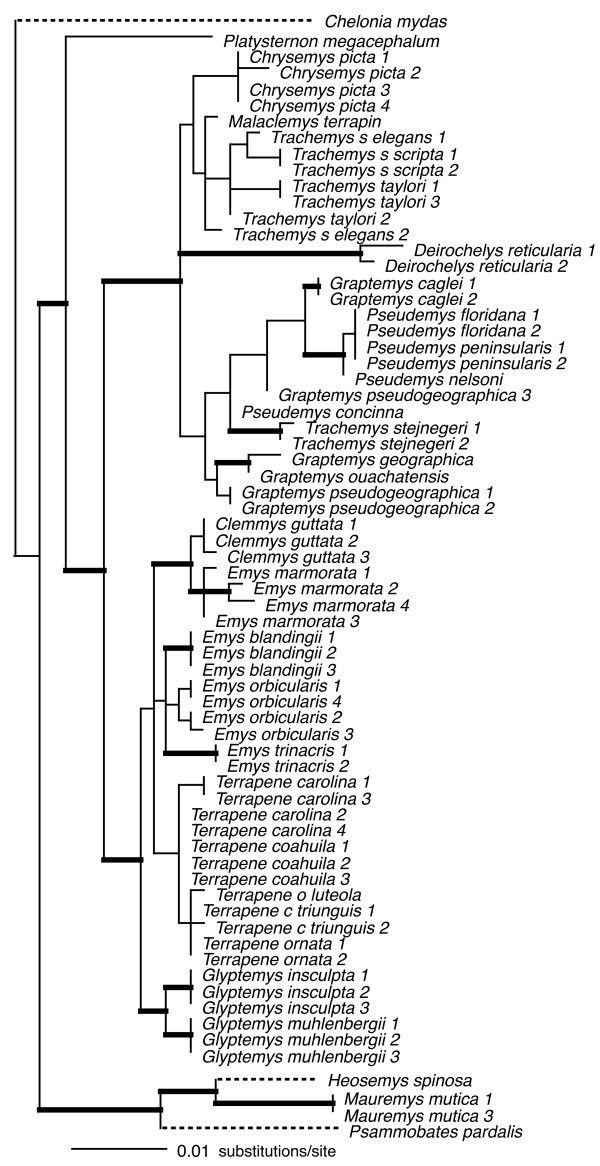
**Maximum-likelihood phylogeny of the 69-taxon HNF-1α data set**. This data set was composed of up to 768 bp. Among the ingroup, 72 characters were parsimony-informative. Estimated ML model parameters conform to the GTR+G model of sequence evolution. -ln *L *= 2533.85397, rate matrix: A-C = 0.70584, A-G = 2.497433, A-T = 0.273807, C-G = 0.59001, C-T = 2.972687, G-T = 1. Base frequencies: A = 0.28, C = 0.23, G = 0.22, and T = 0.27, and γ-shape parameter = 0.670155. Thick branches indicate nodes with ≥ .95 BPP and ML and MP bootstrap values ≥ 70.

**Figure 3:**
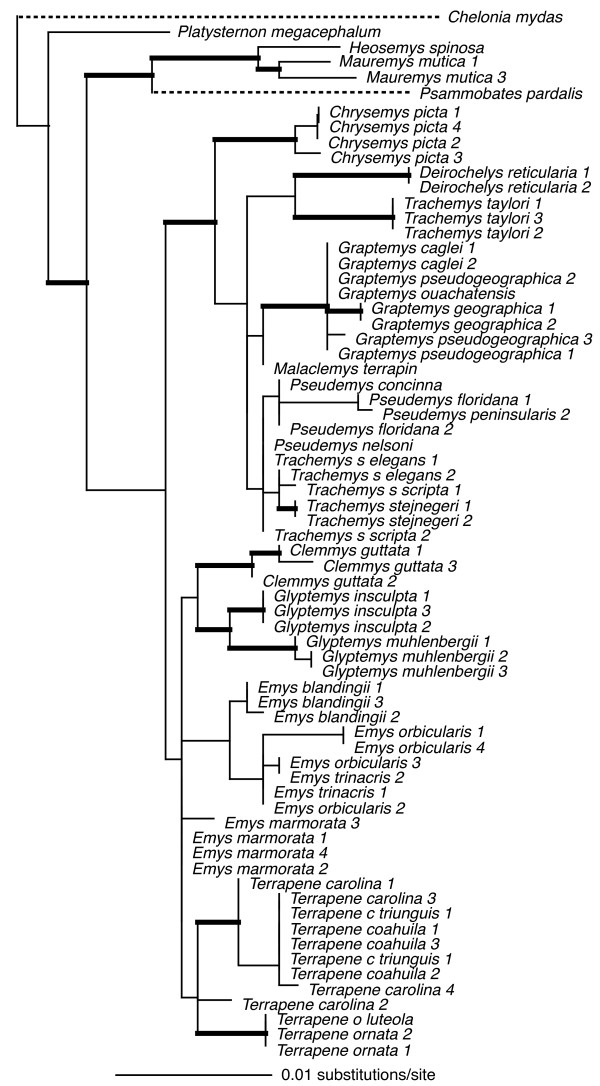
**Maximum-likelihood phylogeny of the 69-taxon R35 data set (978 bp)**. This data set was composed of up to 978 bp. Among the ingroup, 60 characters were parsimony-informative. Estimated ML model parameters conform to the GTR+G model of sequence evolution. -ln *L *= 2571.763999, rate matrix: A-C = 0.939205, A-G = 2.365954, A-T = 0.608604, C-G = 0.875824, C-T = 3.090148, G-T = 1. Base frequencies: A = 0.28, C = 0.18, G = 0.22, and T = 0.32, and γ-shape parameter = 0.582180. Thick branches indicate nodes with ≥ .95 BPP and ML and MP bootstrap values ≥ 70.

**Figure 4:**
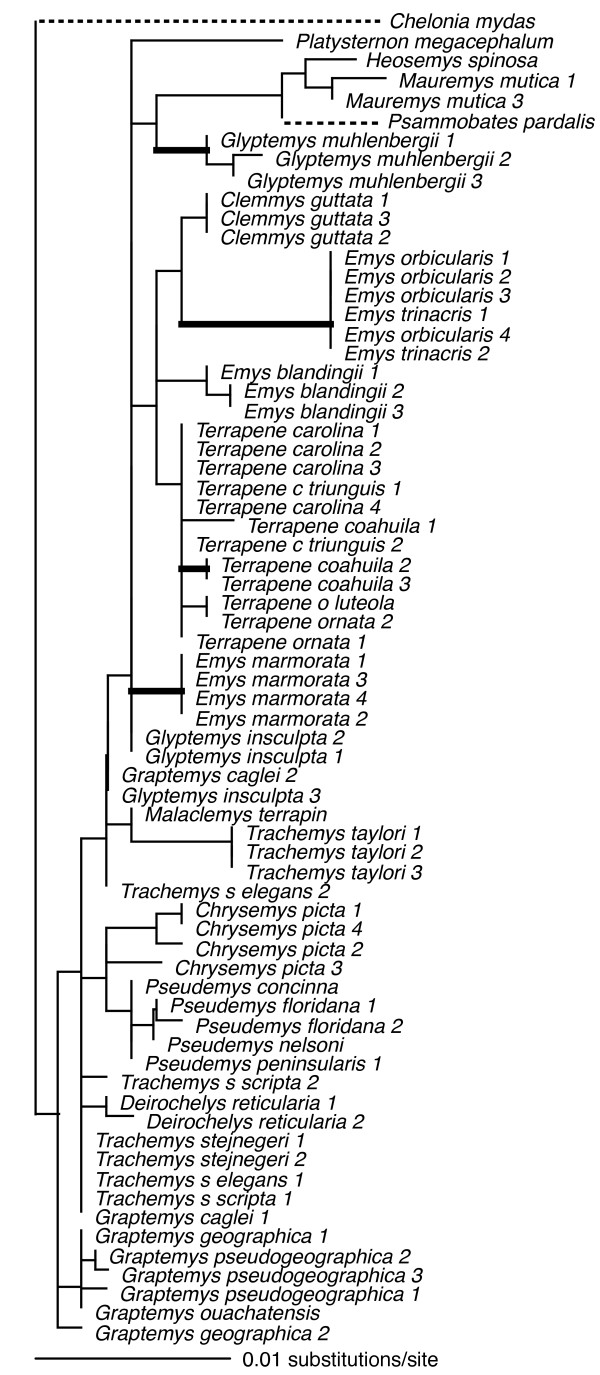
**Maximum-likelihood phylogeny of the 69-taxon RAG-1 data set**. This data set was composed of up to 788 bp. Among the ingroup, 33 characters were parsimony-informative. Estimated ML model parameters conform to the GTR+G+I model of sequence evolution. -ln *L *= 1742.011947, rate matrix: A-C = 0.781722, A-G = 2.299258, A-T = 0.337743, C-G = 0.880761, C-T = 4.054761, G-T = 1. Base frequencies: A = 0.32, C = 0.23, G = 0.22, and T = 0.23, and γ-shape parameter = 0.2.501963. Proportion of invariable sites (I) = 0.462491. Thick branches indicate nodes with ≥ .95 BPP and ML and MP bootstrap values ≥ 70.

**Figure 5:**
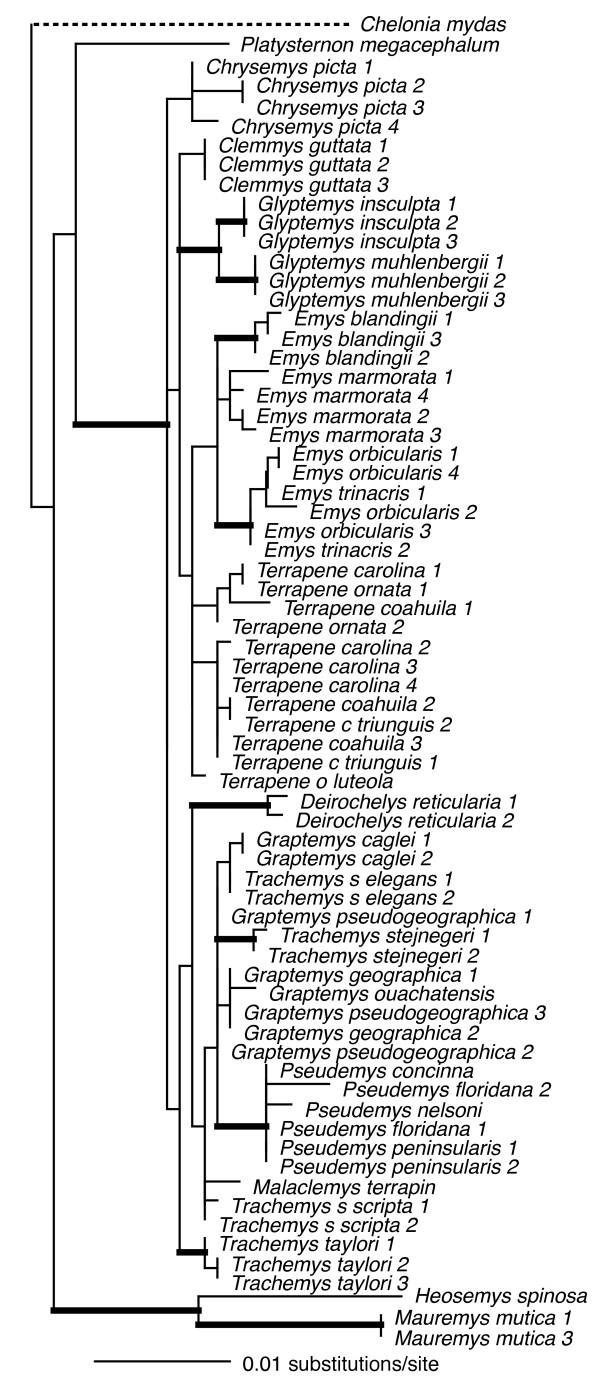
**Maximum-likelihood phylogeny of the 69-taxon RELN data set**. This data set was composed of up to 1104 bp. Among the ingroup, 48 characters were parsimony-informative. Estimated ML model parameters conform to the GTR+G+I model of sequence evolution. -ln *L *= 2847.341935, rate matrix: A-C = 1.068197, A-G = 3.028768, A-T = 0.454495, C-G = 0.612036, C-T = 2.836311, G-T = 1. Base frequencies: A = 0.32, C = 0.17, G = 0.18, and T = 0.33, and γ-shape parameter = 1416.809681. Proportion of invariable sites (I) = 0.482101. Thick branches indicate nodes with ≥ .95 BPP and ML and MP bootstrap values ≥ 70.

**Figure 6:**
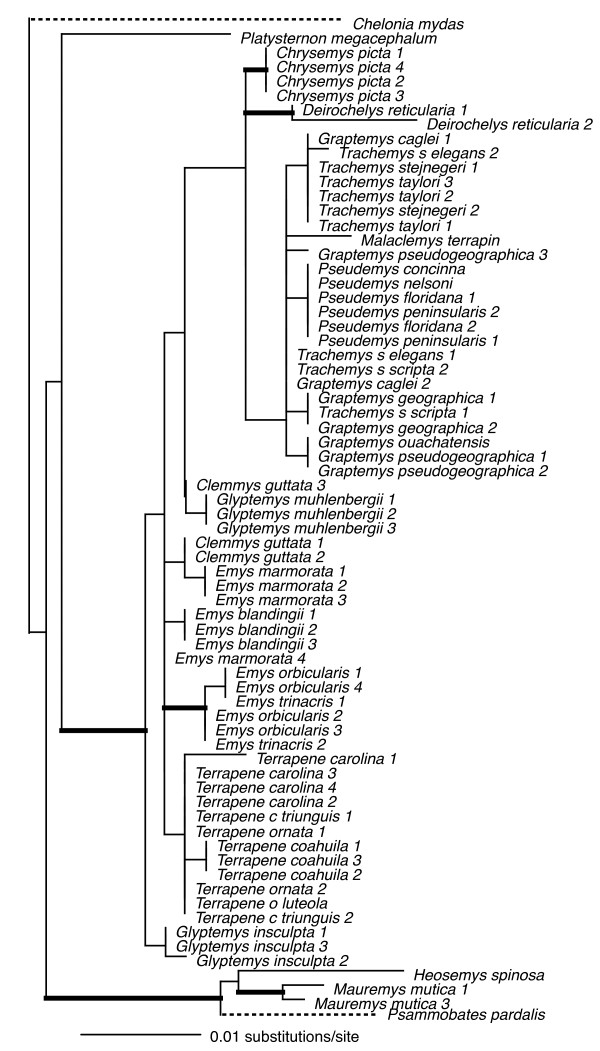
**Maximum-likelihood phylogeny of the 69-taxon TB29 data set**. This data set was composed of up to 590 bp. Among the ingroup, 22 characters were parsimony-informative. Estimated ML model parameters conform to the GTR+G model of sequence evolution. -ln *L *= 1485.985421, rate matrix: A-C = 0.724474, A-G = 2.026332, A-T = 0.393397, C-G = 0.419566, C-T = 1.269648, G-T = 1. Base frequencies: A = 0.31, C = 0.21, G = 0.19, and T = 0.29, and γ-shape parameter = 0.8245. Thick branches indicate nodes with ≥ .95 BPP and ML and MP bootstrap values ≥ 70.

**Figure 7:**
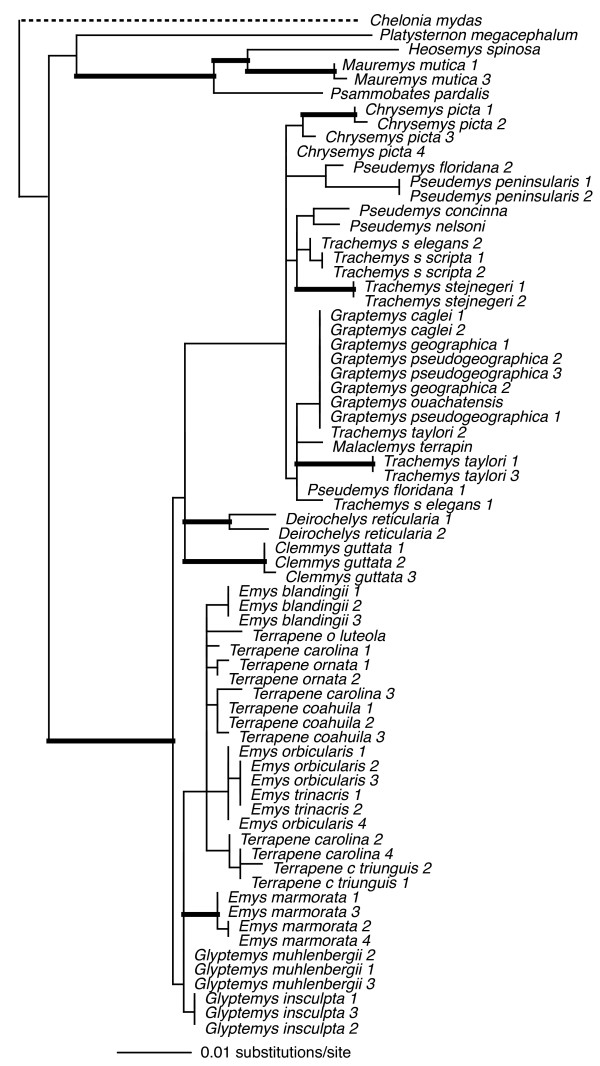
**Maximum-likelihood phylogeny of the 70-taxon TB73 data set**. This data set was composed of up to 668 bp. Among the ingroup, 60 characters were parsimony-informative. Estimated ML model parameters conform to the GTR+G model of sequence evolution. -ln *L *= 2197.656119, rate matrix: A-C = 0.96077, A-G = 2.179468, A-T = 1.20066, C-G = 0.881978, C-T = 2.497604, G-T = 1. Base frequencies: A = 0.29, C = 0.18, G = 0.20, and T = 0.33, and γ-shape parameter = 0.741199. Thick branches indicate nodes with ≥ .95 BPP and ML and MP bootstrap values ≥ 70.

**Figure 8:**
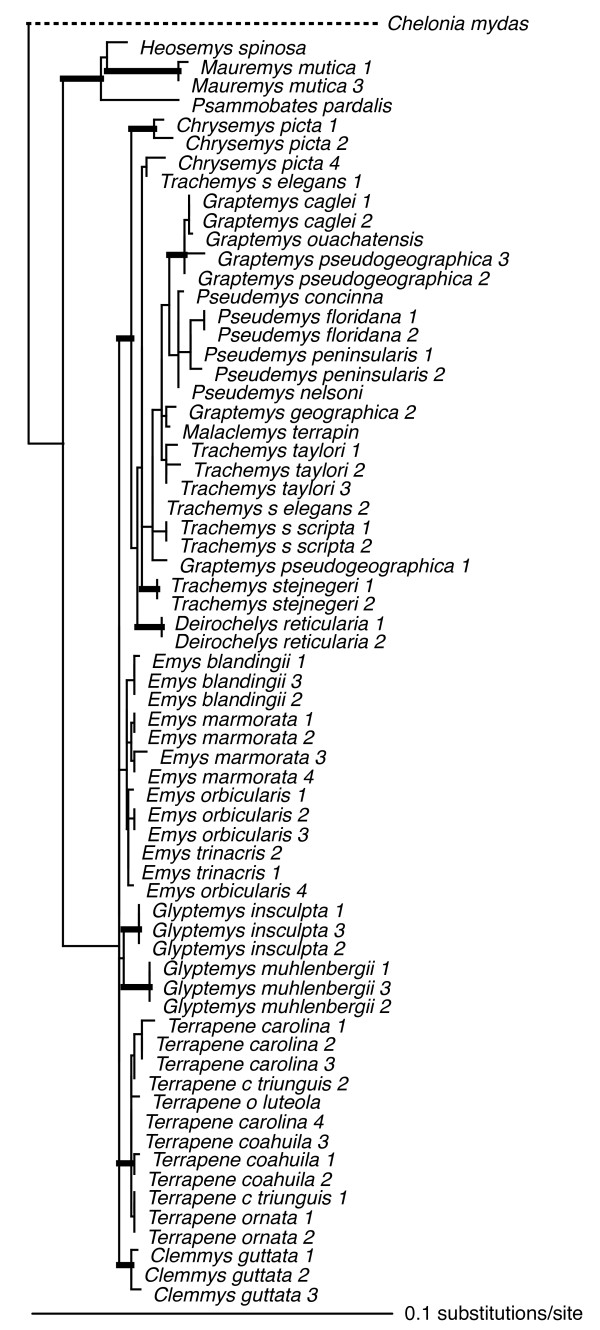
**Maximum-likelihood phylogeny of the 67-taxon TGFB2 data set**. This data set was composed of up to 1078 bp. Among the ingroup, 58 characters were parsimony-informative. Estimated ML model parameters conform to the GTR+G model of sequence evolution. -ln *L *= 3186.405935, rate matrix: A-C = 0.883433, A-G = 2.425602, A-T = 0.769195, C-G = 0.592286, C-T = 1.898448, G-T = 1. Base frequencies: A = 0.29, C = 0.21, G = 0.20, and T = 0.30, and γ-shape parameter = 0.862394. Thick branches indicate nodes with ≥ .95 BPP and ML and MP bootstrap values ≥ 70.

Individual loci ranged in size (590 bp – 1104 bp), and in number of parsimony-informative characters (22 – 72), with the average locus ~850 bp in length, and containing ~50 parsimony-informative characters (see legends Figs 2, 3, 4, 5, 6, 7, 8).

**Figure 9:**
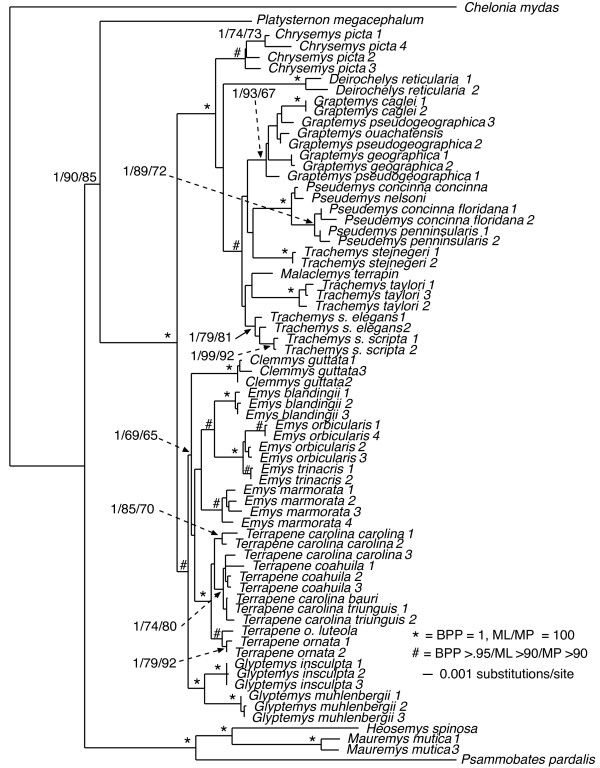
**Maximum-likelihood phylogeny based on the 70-taxon seven-locus nuclear DNA data set**. This data set was composed of up to 5961 bp. Among the ingroup, 350 characters were parsimony-informative. Nuclear loci included HNF-1α, RAG, RELN, R35, TB29, TB73, and TGFB2. Estimated model parameters conform to the GTR+G+I model of sequence evolution. -ln *L *= 17485.22059, rate matrix: A-C = 1, A-G = 2.7416, A-T = 0.6815, C-G = 0.6815, C-T = 2.7416, G-T = 1. Base frequencies: A = 0.30, C = 0.20, G = 0.20, and T = 0.30. Proportion of invariant sites (I) = 0.3263, and γ-shape parameter = 0.9023. Node symbols as in Fig. 1.

To assess the relative phylogenetic performance of individual loci, we generated phylogenies for each locus independently, and under the assumption that current taxonomy is accurate, counted clades recovered from each locus including Emydidae, Deirochelyinae, and Emydinae as well as all genera and species from which we had > 1 sample (Table 1). Phylogenies generated from all loci except RAG-1 recovered the Emydidae as monophyletic with high MP or ML bootstrap support values (Figs 4, 5, 6, 7, 8, 9, Table 1), and the Deirochelyinae was recovered with strong support from HNF-1α, R35, and TGFB2. Similarly, the Emydinae was recovered from HNF-1α, R35, and RELN, but with strong support from HNF-1α only. (Figs 2, 3, 5, 8). Support levels for other clades varied across genes. For example, *Deirochelys *and *E. blandingii *were recovered as monophyletic at all loci, whereas *P. concinna*, and *G. pseudogeographica *as well as two species of *Terrapene *(*carolina, coahuila*) were never recovered as monophyletic (Figs 2, 3, 4, 5, 6, 7, 8, Table 1).

**Table 1:** Table of clades recovered from phylogenetic analyses of individual and concatenated nuclear loci. + indicates clades that were recovered while – indicates clades that were not recovered.

	Locus							Concatenated nuDNA
Clade	HNF-1α	RAG	R35	RELN	TB29	TB73	TGFB	
Emydidae	+	-	+	+	+	+	+	+
Deirochelyinae	+	-	+	-	-	-	+	+
*Chrysemys*	+	+	+	+	+	-	-	+
*Deirochelys*	+	+	+	+	+	+	+	+
*Graptemys*	-	-	+	-	-	-	-	+
*caglei*	+	-	-	+	-	-	-	+
*geographica*	*	-	+	-	+	-	-	+
*pseudogeographica*	-	-	-	-	-	-	-	-
*Pseudemys*	-	+	-	+	+	-	+	+
*concinna*	-	-	-	-	-	-	-	-
*peninsularis*	-	-	-	-	-	-	-	+
*Trachemys*	-	-	-	-	-	-	-	-
*scripta*	-	-	-	-	-	+	-	+
*stejnegeri*	+	-	+	+	-	+	+	+
*taylori*	-	+	+	+	-	-	+	+
Emydinae	+	-	+	+	-	-	-	+
*Clemmys*	+	+	+	+	-	+	+	+
*Emys*	-	-	-	+	-	-	-	+
*blandingii*	+	+	+	+	+	+	+	+
*marmorata*	+	+	-	+	-	+	+	+
*orbicularis*	+	-	-	-	-	-	-	+
*trinacris*	+	-	-	-	-	-	-	+
*Glyptemys*	+	-	+	+	-	-	-	+
*insculpta*	+	-	+	+	+	+	+	+
*muhlenbergii*	+	+	+	+	+	-	+	+
*Terrapene*	-	-	-	+	+	-	+	+
*carolina*	-	-	-	-	-	-	-	-
*coahuila*	-	-	-	-	-	-	-	-
*ornata*	-	-	+	-	-	-	-	+

Totals	15	8	15	16	9	8	12	25

* The monophyly of *G. geographica *could not be assessed for HNF-1α because we were not able to sequence both specimens for this locus.

### Empirical concatenated nuclear phylogeny

Our concatenated nuDNA data set was composed of seven loci and up to 5961 bp of which 4912 were invariant (or excluded). Among ingroup taxa, 350 of these were parsimony informative. Again, this matrix was mostly complete with ~7% missing data. Fig 9 shows the ML tree with BPP and ML/MP bootstrap support values as indicated. The concatenated nuclear data recovered the Deirochelyinae (inclusive of *D. reticularia*), and the Emydinae as reciprocally monophyletic with strong support from all analytical methods. All genera were monophyletic with strong support except *Trachemys*, and all species except *Graptemys pseudogeographica*, *P. concinna*, *T. carolina*, and *T. coahuila *were monophyletic, mostly with strong support (Fig. 9). However, the branches subtending most genera were relatively short, and intergeneric relationships within subfamilies were mostly unsupported.

### Simulations

**Figure 10:**
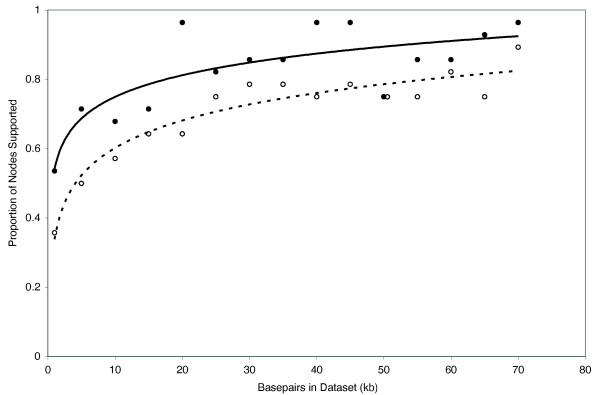
**Maximum likelihood simulations showing proportion of nodes with bootstrap support values of ≥ 70 (filled circles) and ≥ 95 (open circles)**. Due to computational constraints, we analyzed every 5^th ^data set only (i.e. in 5 kb, 10 kb 15 kb etc.) under maximum likelihood.

**Figure 11:**
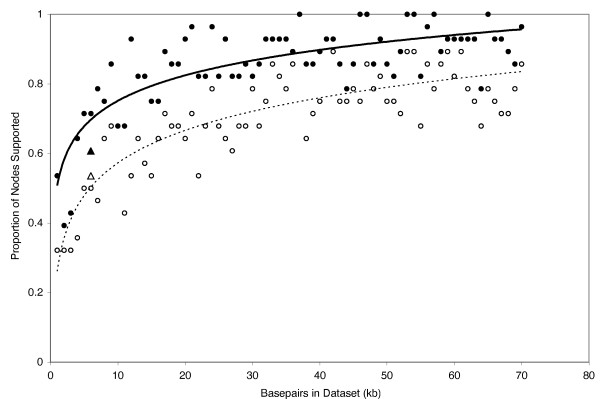
**Maximum parsimony simulations showing proportion of nodes with MP bootstrap support values of ≥ 70 (filled circles) and ≥ 95 (open circles)**. Also shown are support values recovered from analyses of a 31-taxon empirical nuDNA data set (filled triangle = ≥ 70, open = ≥ 95).

**Figure 12:**
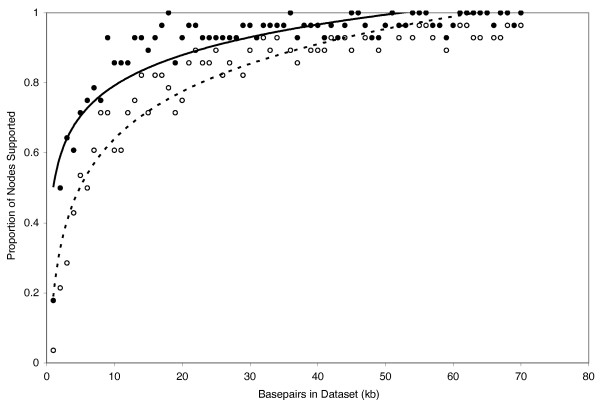
**Maximum parsimony simulations of the RAG-1 data set showing proportion of nodes with MP bootstrap support values of ≥ 70 (filled circles) and ≥ 95 (open circles)**.

Results from the ML simulations (Fig. 10) were qualitatively very similar to our MP simulation results (Fig. 11) in that an increase in data generally resulted in an overall increase in support values. Bootstrap support values of ≥ 95 for all nodes were eventually reached in some datasets except for the ML simulations, where the maximum proportion of nodes recovered was 96% and 90% at bootstrap support levels of ≥ 70 and ≥ 95, respectively (Fig. 10). However, the single-model simulation based on RAG-1 only (Fig. 12) reached higher overall levels of support with less data than did the "full" simulations (i.e. those based on all seven nuclear loci). For example, of the 70 data sets from the single-locus simulation, 57 had > 90% of nodes supported at the ≥ 70 bootstrap support level. In contrast, 28 of the 70 data sets from the full simulation had > 90% of nodes supported at the ≥ 70 bootstrap support level. Results were more one-sided for the ≥ 95 bootstrap support level where 32 of 70 data sets from the single-locus simulation, but only 2 of 70 data sets from the full simulation had > 90% of nodes supported at the ≥ 95 bootstrap support level (Figs 11, 12).

**Figure 13:**
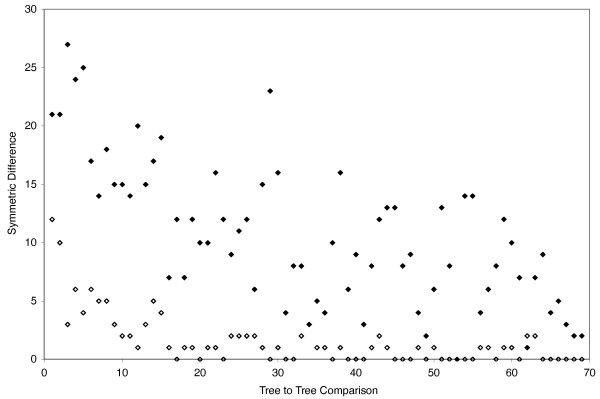
**Symmetric tree distances generated from the single-gene simulation (open diamonds), and the full MP simulation (filled diamonds)**.

To examine these effects more quantitatively, we 1) show symmetric tree distances plotted as a function of total data for 1 kb incremental increases from 1–70 kb of simulated data, and 2) compare MP phylogenies generated from empirical data with those generated from simulated data. The key result from the symmetric tree distances plots is that the symmetric tree distances are much lower for single versus multiple gene simulations. For example, the average symmetric tree distance for the single-locus simulation (1.5) was almost an order of magnitude lower than the average from the full simulation (10.7) (Fig. 13). In addition, trees generated from simulated data were also similar to those from our empirical data. To compare trees from simulated vs empirical data, we performed an MP bootstrap analysis on the empirical data (31-taxon, 5974 bp), and compared these trees to results from 6000 bp of simulated data (trees not shown). Support values from the empirical data were slightly lower than those from simulated data where 61% of nodes were recovered at the ≥ 70 bootstrap support level, compared to 75% and 71% of nodes for the single-locus and full simulations, respectively. However, at the ≥ 95 support level about 50% of nodes were recovered from analyses of empirical and simulated data (Fig. 11).

## Discussion

Our results speak both to general issues in phylogenetic data requirements and specific progress in the phylogeny of emydid turtles. Overall, our results argue strongly for the insights that can be gained from a combination of multi-gene empirical results which summarize our current state of phylogenetic knowledge, and biologically realistic simulations to gain a sense of the data required to further clarify these phylogenetic results.

### Phylogenetic resolution: empirical and simulation results

At least for problems of the size and complexity of Emydidae, ~6 kb of nuclear sequence data were clearly insufficient to recover well-supported relationships among many genera or species. Roughly half of the nodes were recovered with ≥ 95 MP bootstrap support (Fig. 9), and those nodes were spread across both shallow and deep nodes of the tree. About 1 kb of mitochondrial DNA yielded similar support levels (Fig. 1). However, except for species and genus monophyly, there was only a single relationship (*Glyptemys *as sister to the remaining emydines) shared by both analyses. Although it remains unclear whether this incongruence has a biological basis (i.e. introgression/hybridization), is due to incomplete lineage sorting, or results from some combination of factors, it certainly confirms the growing suspicion that phylogenetic conclusions and taxonomic changes based purely on mitochondrial gene trees may often be premature, and that multiple markers are required for this level of analysis. However, determining how much data might be required remains challenging.

Overall, our simulations suggest that, although the percentage of nodes supported is always a decreasing function of additional data analyzed, relatively high overall support values can probably be obtained with moderate amounts of data. However, previous simulations based on single (or a few) loci–because they ignore variation among gene trees–probably underestimate the amount of data necessary for recovering robust phylogenies. For example, with 20 kb sequence data, 82% and 67% of nodes were recovered with bootstrap support values of ≥ 70 and ≥ 95, respectively, but increasing the data sampling by 50% had little effect on support values. With 30 kb, 87% and 72% of nodes were recovered with bootstrap support values of ≥ 70 and ≥ 95, respectively; with 40 kb, each increased by another percent or two. Thus, all else being equal, there was relatively little gain in overall number of supported nodes after the first ~24 loci (assuming a standard locus is about 850 bp). Rokas et al. (2003) found empirically that data from ≥ 20 genes was sufficient to recover the phylogeny of *Saccharomyces *yeast species with strong support, based on subsamples of their 106-locus dataset. Although ~24 independent nuclear markers currently stretches the limits of most non-model organism datasets, this need not be the case in the near future. As additional genomic resources become available, assembling 24 or more markers should become feasible for many metazoan, plant, and fungal taxa, using either traditional universal-primer [14,47,11,13] or more clade-restrictive strategies [12] for primer development.

Among our empirical loci, HNF-1α, was one of the shortest, but had the most parsimony-informative characters of all loci (see Fig. legends). Therefore we wanted to determine if HNF-1α (or any other locus) might have had a disproportionate impact on our multi-locus simulations. For example, our a priori expectation is that if HNF-1α were driving the simulations, then simulated trees should be similar or identical to the empirical tree generated from HNF-1α. We tested this prediction using the SH test. To carry this out, we compared the 70 kb MP 50% majority rule consensus tree from the simulations with empirical 50% majority rule consensus trees generated from each locus. The 70 kb MP tree had the lowest -ln *L *score and trees generated from all seven empirical loci were significantly longer than the 70 kb MP tree (*P *≤ 0.048). Thus, the simulations did not appear to be overly influenced by any one marker.

As in previous simulation studies, our single-locus simulation results (based on RAG-1) recovered relatively high support values and low symmetric distances compared to the full simulations (Figs 12, 13). The RAG-1 data were simulated using a single input tree and model of molecular evolution (see below). In contrast, each dataset from the full simulation was compiled from markers drawn independently from the pool of simulated data. Consequently, our nuDNA data was simulated from a minimum of one input tree/model of molecular evolution up to a maximum of 70 input trees, and seven models of molecular evolution. Thus, the high variance in support values and symmetric tree distances among data sets from the full simulation suggest that the phylogenetic performance of data drawn from individual loci may be conveying a somewhat false sense of encouragement compared to more thorough multi-gene simulations. In other words, a well-supported tree generated from a single locus, either in a simulation or empirical framework, does not necessarily mean that the organismal phylogeny is known with confidence. Essentially, the largely stochastic variance among genes and their associated gene trees is never challenged by independent data in single gene analyses, which can leave one with greater support for idiosyncratic gene tree results than one might obtain based on a more comprehensive sampling of gene tree histories, and this was born out by the SH tests. The message is clear–empirical studies of long reads from single genes, and simulations based on single genes, will often yield overly optimistic views of the certainty of organismal phylogenies.

### Emydid phylogeny

Generally, our empirical phylogenetic results were in line with our *a priori *expectations in that the mtDNA tree was well-resolved, well supported, and consistent with previous mtDNA results while our nuDNA tree was not as well supported, and contained relatively short branches among most emydine and deirochelyine genera. Direct comparisons of our mtDNA and nuDNA-based trees was complicated by differences in number of terminals, but at the mitochondrial level (excluding outgroups) 86% of nodes (19/22) were well supported from at least one analytical method, but only about half (11/23) of interspecific nodes were supported based on nuDNA (Figs 1, 9). Both datasets agree on the monophyly of most genera (some of which contain only a single species), but not on relationships among those genera. For example, the nuclear dataset strongly supported the monophyly of the traditionally-recognized subfamily Deirochelyinae; within it, a *Graptemys-Pseudemys-Malaclemys-Trachemys *clade was the only strongly supported node. Both of these groups were rejected by the mtDNA dataset (SH test, *P *< 0.01). Similarly, the nuclear dataset supported the monophyly of the traditional Emydinae, the sister-group relationship of *Glyptemys *to all remaining emydines, *Clemmys *as sister group to the *Emys-Terrapene *clade, and *E. marmorata *as sister to the remaining species of *Emys*; of these, only the position of *Glyptemys *was also supported by the mtDNA analysis. Further, a more detailed examination of the *Emys *species relationships revealed at least in that case, that the mtDNA phylogeny was most likely the result of an ancient hybridization and mitochondrial gene capture event [48].

## Conclusion

Overall, short branches among emydine and deirochelyine genera, particularly for nuclear genes, suggests that recovering relationships among genera and species of this clade of turtles will continue to be a difficult problem. We are making important progress towards our understanding of emydid phylogeny and taxonomy, both in terms of species boundaries and interspecific phylogeny, but that progress has been slow and clearly requires both new data and new approaches. In particular, the exceedingly problematic genus *Pseudemys*, which has been a source of taxonomic uncertainty for over 150 years, while the box turtles (*Terrapene*) and map turtles (*Graptemys*) all remain problematic both in terms of species delimitation and within-genus interspecific phylogenetics [31-35]. All of these groups may well require a more population genetic approach to fully resolve. On the other hand, each of the three *Trachemys *species examined here were monophyletic with strong support based on both mtDNA and nuDNA sequences, although relationships among them remain obscure.

Incongruence between mtDNA vs nuDNA phylogenies is not uncommon, and generating additional data is a logical step towards understanding this incongruence. Although simulations do not inform us as to the causes of among-tree disagreements, they can be useful for determining the utility of generating additional empirical data to solve remaining, difficult parts of phylogenies. In order for these simulations to provide accurate expectations for future studies, they should embrace realistic levels of among-gene-tree variation by simulating many genes rather than being based on one or a few empirical markers.

## Methods

### Taxon Sampling

Our analysis incorporated a total of 70 individuals including 64 ingroup, and six outgroup taxa representing all genera and 25 of 48 emydid species (Additional file 1). Our analyses included > two individuals of each species except *Malaclemys terrapin*. Of the two recognized subfamilies of emydids, we were missing one of the eleven species of Emydinae (the Mexican box turtle *Terrapene nelsoni*) and several species each from the diverse deirochelyine genera *Graptemys*, *Pseudemys *and *Trachemys*.

### Data Sampling

We downloaded 103 sequences from GenBank, most of which were generated previously by us (Spinks and Shaffer in press). New sequences generated for this study were generally from the same individual specimen represented in GenBank (Additional file 1). Genomic DNA was extracted from blood or other soft tissue samples using a salt extraction protocol [49], and sequences from multiple markers were almost always generated from the same individual (Additional file 1). We sequenced fragments of one mitochondrial gene and up to seven nuclear loci. PCR products were amplified using 15–20 μL volume Taq-mediated reactions with an initial heating of 95° for 60 seconds, followed by 35 cycles of denaturation at 94°C; 30 seconds, annealing at 58°C-65°C; 30 seconds, and extension at 72°C; 45–90 seconds followed by a final extension of 72° for 10 minutes. Annealing temperatures, extension times, and primer sequences can be found in references following each locus. For mtDNA, we used cytochrome *b *(cyt*b*, [50]) while our nuclear DNA (nuDNA) included intron 2 of the hepatocyte nuclear factor 1α (HNF-1α, [51]) the nuclear recombination activase gene 1 (RAG-1, [52]), intron 61 of the Reelin gene (RELN, [53]), intron 1 of the fingerprint protein 35 (R35, [54]), intron 5 of the transforming growth factor beta-2 (TGFB2, [51]), and two anonymous nuclear loci: TB29 and TB73 [12]. All PCR products were sequenced in both directions on ABI 3730 automated sequencers at the UC Davis Division of Biological Sciences sequencing facility .

### Phylogenetic Analyses

The cyt*b *sequences were translated using MacClade 4.06 [55] to check for potential sequencing errors and orthology problems, including nuclear mitochondrial pseudogenes (numts). Sequences were initially aligned using MacClade 4.06 with final editing of the alignments by hand in PAUP* 4.0b10 [56], and the alignment was deposited in TreeBase [, accession # S2303]. We performed phylogenetic analyses on mitochondrial and nuclear loci separately. Phylogenetic analyses of the mitochondrial and empirical concatenated nuclear sequence data sets were performed under ML, MP and Bayesian Inference. ML and MP analyses were performed using PAUP* 4.0b10 [56] with ten random stepwise heuristic searches and tree bisection-reconnection (TBR) branch swapping (for MP), or subtree pruning-regrafting (SPR) branch swapping (for ML). Model parameters for ML and Bayesian analyses were estimated in PAUP* 4.0b10, and selected under the Akiake Information Criterion (AIC). Modeltest 3.06 [57] was used to report model parameters for use in ML analyses. We bootstrapped each data set with 100 pseudoreplicates [58], limiting each ML bootstrap replicate to one hour of computation time. For individual nuclear loci, we used RaxML [59] and MrBayes through the CIPRES web portal  to carry out ML bootstrap and Bayesian analyses.

For the mitochondrial and concatenated nuDNA analyses, we used MrBayes V3.1.1 [60,61] to perform partitioned model Bayesian analyses. The mitochondrial sequences were partitioned by codon position while the nuDNA sequences were partitioned by locus. All Bayesian analyses were performed with two replicates and four chains for 10^5 ^generations. Chains were sampled every 10^3 ^generations, and stationarity was determined when the -log likelihood (-ln *L*) scores plotted against generation time visually reached a stationary value, and when the potential scale reduction factor (PSRF) equaled 1. Trees sampled prior to stationarity were discarded as burn-in.

### Simulations

Our simulation approach was of the data-growing type. Importantly, we generalized our simulation procedure in such a way as to provide a more biologically realistic estimate. In particular, we increased the among-gene variance of the simulated data by incorporating variation in models of molecular evolution, model parameter values, and gene tree topologies derived from our empirical nuclear sequence data set (see below).

**Figure 14:**
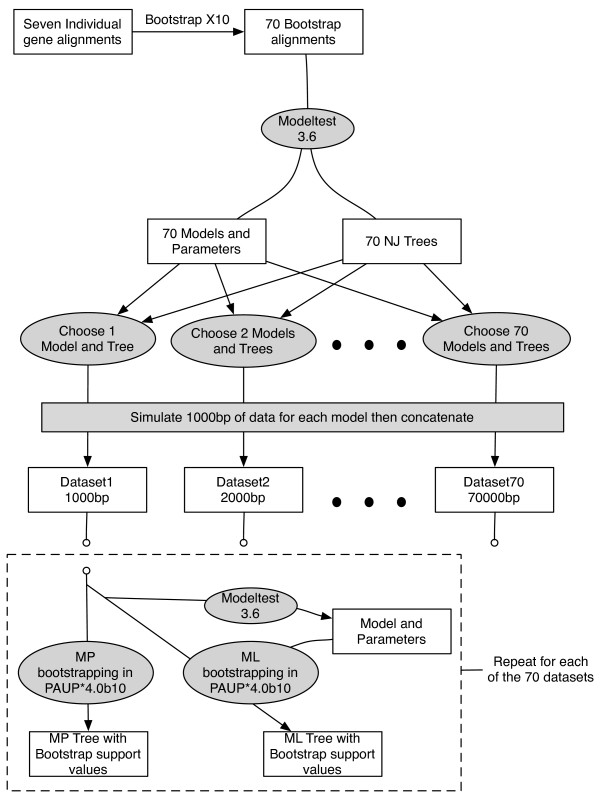
**Flow chart detailing methodology for generating simulated data sets**. See text for full description.

Our simulation procedure is shown as a flow chart in Fig 14. For the simulations, we did not include the mitochondrial sequences since they are not representative of loci sampled from the nuclear genome. We assembled a 31-taxon data set that included all seven nuclear loci and one exemplar of each emydid species plus all outgroup taxa. *Graptemys geographica *had a great deal of missing data, and so was excluded from the simulation analysis. For each data partition we produced 10 nonparametric bootstrap replicated datasets using the Seqboot module of the Phylip 3.66 package [62], yielding 70 unique datasets. For each of these datasets, we selected the best fitting model of molecular evolution using Modeltest 3.6 [57] and selected appropriate models via the AIC. In addition, as part of the model selection procedure the Modeltest 3.6 program uses a Modelblock batch file (executed in PAUP* 4.0b10). This batch file generated neighbor-joining (NJ) trees that were used in the model determination procedure. We modified the Modelblock batch file to save these NJ starting trees from each of the 70 simulated data sets. This yielded a pool of 70 models of nucleotide sequence evolution plus their corresponding parameter estimates and NJ trees. The nonparametric bootstrapping step was employed in order to generate a large number of models and trees that were similar to the seven models and trees inferred from the empirical data, and yet incorporated a degree of variation as might be observed if one were to sample additional similar loci.

Next, we constructed simulated datasets by selecting a model, and its corresponding NJ tree at random (with replacement) from the pool of 70, and used Seq-Gen1.3.2 [63] to simulate 1000 nucleotide characters for each model/tree combination. This process was repeated and each simulated block of nucleotide characters was concatenated to produce simulated datasets ranging in size from 1000 bp (one model/tree only) to 70000 bp (70 models/trees). In addition, each dataset was simulated independently with a new model drawn from the pool for each subsequent data set (i.e. the 17^th ^dataset did **not **consist of the 16^th ^dataset with 1000 more base pairs added).

Phylogenies and bootstrap support values (100 pseudoreplicates) were generated using PAUP* 4.0b10 for each simulated dataset under MP. However, due to computational constraints, we analyzed every fifth data set only under ML (i.e. 5000 bp, 10000 bp, 15000 bp, etc.). MP bootstrap replicates were carried out using random sequence addition and TBR branch swapping, with the multiple trees option in effect. For ML bootstrap analyses, we chose a new model of molecular evolution for each simulated dataset using PAUP* 4.0b10, and Modeltest 3.6 with AIC-selected parameters, and employed the same heuristic search settings as in the MP bootstrap analyses. Support values for each simulated phylogeny were determined by counting the total number of nodes in each tree that were supported at the ≥ 70 and ≥ 95 level. To quantitatively compare trees among simulations, we used the symmetric tree distance [64,65] (symmetric difference test implemented in PAUP* 4.0b10). Trees were compared sequentially such that the tree generated from the 1000 bp data set (tree 1) was compared to the tree generated from 2000 bp (tree 2) then tree 2 was compared to tree 3 and so forth.

Our empirical loci varied in length and number of parsimony-informative characters, thus our multi-locus simulation might have been overly influenced by one or a few markers. For example, the average locus was ~850 bp, and contained 50 parsimony informative characters, but at 768 bp, HNF-1α had the most parsimony informative characters (72) of any locus. Therefore, HNF-1α might have had a disproportionate influence on our simulations. We used SH tests (conducted in PAUP* 4.0b10) to assess the impact of this among-locus variation on our simulations. Based on the empirical 31-taxon, seven-locus data set, we compared the 50% majority rule consensus MP tree generated from the 70 kb simulated data to the 50% majority rule consensus MP trees generated from each empirical locus, but with the trees pruned of all but a set of 29 taxa common to all loci. Our reasoning was that if a single locus were driving our simulations then that gene tree would not be significantly different from the tree based on 70 kb of simulated data.

Finally, we compared our simulation procedure to previous methods where data were simulated from a single locus. To carry this out, we repeated our simulation strategy, but used the single empirical model/NJ tree from the RAG-1 locus as input parameters in Seq-Gen to generate 70 datasets ranging from 1000 bp to 70000 bp. We chose RAG-1 since it is one of the most commonly employed phylogenetic markers for vertebrate taxa.

The simulations and tallies of support values were largely automated using a system of PERL, BASH, and R scripts, as well as several PAUP batch files (available from RCT's website, ).

## Authors' contributions

PQS, RCT and HBS developed the project and designed the simulations. PQS and GAL collected the data. RCT performed the simulations. All authors contributed to writing the ms. All authors read and approved the final manuscript.

## Supplementary Material

Additional File 1 **Sample identification, and GenBank accession numbers for all sequences used in this study.** All of the GenBank Accession numbers used in this analysis are listed in this table. Click here for file
