# Supplementary material for: Assessing what is needed to resolve a molecular phylogeny: simulations and empirical data from emydid turtles
Source: BMC Evol Biol. 2009 Mar 12;9:56. doi: 10.1186/1471-2148-9-56 (PMC2660309; doi:10.1186/1471-2148-9-56)
Supplement: Additional File 1 — Sample identification, and GenBank accession numbers for all sequences used in this study. All of the GenBank Accession numbers used in this analysis are listed in this table. [file 1471-2148-9-56-S1.doc]

Additional file 1. Sample identification, and GenBank accession numbers for all sequences used in this study.

|  |  | **GenBank accession #'s** | | | | | | | |
| --- | --- | --- | --- | --- | --- | --- | --- | --- | --- |
| **Tissue #** | **Species** | **Cyt*b*** | **HNFL** | **RELN** | **R35** | **RAG-1** | **TB29** | **TB73** | **TGFB2** |
| HBS 23173 | *Chrysemys picta* 1 | FJ770586 | FJ770625 | FJ770758 | FJ770669 | FJ770714 | FJ770804 | FJ770850 | FJ770896 |
| HBS 26210 | *Chrysemys picta* 2 | FJ770587 | FJ770626 | FJ770759 | FJ770670 | FJ770715 | FJ770805 | FJ770851 | FJ770897 |
| HBS 27134 | *Chrysemys picta* 3 | FJ770588 | FJ770627 | FJ770760 | FJ770671 | FJ770716 | FJ770806 | FJ770852 | NA |
| HBS 27448 | *Chrysemys picta* 4 | FJ770589 | FJ770628 | FJ770761 | FJ770672 | FJ770717 | FJ770807 | FJ770853 | FJ770898 |
| MVZ 175961 | *Clemmys guttata* 2 | FJ770590 | FJ770629 | FJ770762 | FJ770673 | FJ770718 | FJ770808 | FJ770854 | FJ770899 |
| FMNH 265273 | *Clemmys guttata* 3 | FJ770591 | FJ770630 | FJ770763 | FJ770674 | FJ770719 | FJ770809 | FJ770855 | FJ770900 |
| MVZ 137744 | *Dierochelys r. reticularia* 1 | FJ770592 | FJ770631 | FJ770764 | FJ770675 | FJ770720 | FJ770810 | FJ770856 | FJ770901 |
| HBS 108680 | *Dierochelys r. chrysea* 2 | FJ770593 | FJ770632 | FJ770765 | FJ770676 | FJ770721 | FJ770811 | FJ770857 | FJ770902 |
| IPMB 4706 | *Emys trinacris* 1 | AJ131415 | FJ770633 | FJ770766 | FJ770677 | FJ770722 | FJ770812 | FJ770858 | FJ770903 |
| IPMB 4707 | *Emys trinacris* 2 | AJ131415 | FJ770634 | FJ770767 | FJ770678 | FJ770723 | FJ770813 | FJ770859 | FJ770904 |
| HBS 108712 | *Glyptemys insclpta* 2 | FJ770594 | FJ770635 | FJ770768 | FJ770679 | FJ770724 | FJ770814 | FJ770860 | FJ770905 |
| HBS 108711 | *Glyptemys insclpta* 3 | FJ770595 | FJ770636 | FJ770769 | FJ770680 | FJ770725 | FJ770815 | FJ770861 | FJ770906 |
| HBS 108716 | *Glyptemys muhlenbergii* 2 | FJ770596 | FJ770637 | FJ770770 | FJ770681 | FJ770726 | FJ770816 | FJ770862 | FJ770907 |
| HBS 108717 | *Glyptemys muhlenbergii* 3 | FJ770597 | FJ770638 | FJ770771 | FJ770682 | FJ770727 | FJ770817 | FJ770863 | FJ770908 |
| HBS 108718 | *Graptemys caglei* 1 | NA | FJ770639 | FJ770772 | FJ770683 | FJ770728 | FJ770818 | FJ770864 | FJ770909 |
| HBS 108719 | *Graptemys caglei* 2 | NA | FJ770640 | FJ770773 | FJ770684 | FJ770729 | FJ770819 | FJ770865 | FJ770910 |
| HBS 23396 | *Graptemys geographica* 1 | NA | FJ770641 | FJ770774 | FJ770685 | FJ770730 | FJ770820 | FJ770866 | NA |
| HBS 23397 | *Graptemys geographica* 2 | FJ770598 | NA | FJ770775 | FJ770686 | FJ770731 | FJ770821 | FJ770867 | FJ770911 |
| HBS 23347 | *Graptemys ouachatensis* | FJ770599 | FJ770642 | FJ770776 | FJ770687 | FJ770732 | FJ770822 | FJ770868 | FJ770912 |
| HBS 11150 | *Graptemys p. koni* 1 | FJ770600 | FJ770643 | FJ770777 | FJ770688 | FJ770733 | FJ770823 | FJ770869 | FJ770913 |
| HBS 23217 | *Graptemys pseudogeographica* 3 | FJ770601 | FJ770644 | FJ770778 | FJ770689 | FJ770734 | FJ770824 | FJ770870 | FJ770914 |
| MVZ 137745 | *Malaclemys terrapin* | FJ770602 | FJ770645 | FJ770779 | FJ770690 | FJ770735 | FJ770825 | FJ770871 | FJ770915 |
| HBS 23325 | *Pseudemys c. concinna* | FJ770603 | FJ770646 | FJ770780 | FJ770691 | FJ770736 | FJ770826 | FJ770872 | FJ770916 |
| HBS 108683 | *Pseudemys c. floridana* 1 | FJ770604 | FJ770647 | FJ770781 | FJ770692 | FJ770737 | FJ770827 | FJ770873 | FJ770917 |
| HBS 108682 | *Pseudemys c. floridana* 2 | FJ770605 | FJ770648 | FJ770782 | FJ770693 | FJ770738 | FJ770828 | FJ770874 | FJ770918 |
| HBS 108599 | *Pseudemys nelsoni* | NA | FJ770649 | FJ770783 | FJ770694 | FJ770739 | FJ770829 | FJ770875 | FJ770919 |
| HBS 108722 | *Pseudemys penninsularis* 1 | FJ770606 | FJ770650 | FJ770784 | FJ770695 | FJ770740 | FJ770830 | FJ770876 | FJ770920 |
| HBS 108723 | *Pseudemys penninsularis* 2 | FJ770607 | FJ770651 | FJ770785 | NA | NA | FJ770831 | FJ770877 | FJ770921 |
| HBS 27355 | *Terrapene c. carolina* 2 | FJ770608 | FJ770652 | FJ770786 | FJ770696 | FJ770741 | FJ770832 | FJ770878 | FJ770922 |
| HBS 27366 | *Terrapene c. carolina* 3 | FJ770609 | FJ770653 | FJ770787 | FJ770697 | FJ770742 | FJ770833 | FJ770879 | FJ770923 |
| HBS 108689 | *Terrapene c. bauri* 4 | FJ770610 | FJ770654 | FJ770788 | FJ770698 | FJ770743 | FJ770834 | FJ770880 | FJ770924 |
| HBS 108677 | *Terrapene coahuila* 2 | FJ770611 | FJ770655 | FJ770789 | FJ770699 | FJ770744 | FJ770835 | FJ770881 | FJ770925 |
| HBS 108678 | *Terrapene coahuila* 3 | FJ770612 | FJ770656 | FJ770790 | FJ770700 | FJ770745 | FJ770836 | FJ770882 | FJ770926 |
| HBS 108701 | *Terrapene o. luteola* | FJ770614 | FJ770657 | FJ770791 | FJ770701 | FJ770746 | FJ770837 | FJ770883 | FJ770927 |
| HBS 27365 | *Terrapene c. triunguis* 1 | FJ770615 | FJ770658 | FJ770792 | FJ770702 | FJ770747 | FJ770838 | FJ770884 | FJ770928 |
| HBS 27385 | *Terrapene c. triunguis* 2 | FJ770616 | FJ770659 | FJ770793 | FJ770703 | FJ770748 | FJ770839 | FJ770885 | FJ770929 |
| HBS 35662 | *Terrapene ornata* 2 | FJ770613 | FJ770660 | FJ770794 | FJ770704 | FJ770749 | FJ770840 | FJ770886 | FJ770930 |
| HBS 27243 | *Trachemys s. elegans* 2 | FJ770617 | FJ770661 | FJ770795 | FJ770705 | FJ770750 | FJ770841 | FJ770887 | FJ770931 |
| HBS 108688 | *Trachemys s. scripta* 1 | FJ770618 | FJ770662 | FJ770796 | FJ770706 | FJ770751 | FJ770842 | FJ770888 | FJ770932 |
| HBS 108687 | *Trachemys s. scripta* 2 | FJ770619 | FJ770663 | FJ770797 | FJ770707 | FJ770752 | FJ770843 | FJ770889 | FJ770933 |
| HBS 108728 | *Trachemys stejnegeri* 1 | FJ770620 | FJ770664 | FJ770798 | FJ770708 | FJ770753 | FJ770844 | FJ770890 | FJ770934 |
| HBS 108729 | *Trachemys stejnegeri* 2 | FJ770621 | FJ770665 | FJ770799 | FJ770709 | FJ770754 | FJ770845 | FJ770891 | FJ770935 |
| HBS 108673 | *Trachemys taylori* 1 | FJ770622 | FJ770666 | FJ770800 | FJ770710 | FJ770755 | FJ770846 | FJ770892 | FJ770936 |
| HBS 108679 | *Trachemys taylori* 2 | FJ770623 | FJ770667 | FJ770801 | FJ770711 | FJ770756 | FJ770847 | FJ770893 | FJ770937 |
| HBS 108674 | *Trachemys taylori* 3 | FJ770624 | FJ770668 | FJ770802 | FJ770712 | FJ770757 | FJ770848 | FJ770894 | FJ770938 |
| HBS 16391 | *Clemmys guttata* 1 | EU787026 | EU787083 | EU787297 | EU787165 | EU787247 | EU787273 | EU787380 | EU787225 |
| HBS 23408 | *Emys blandingii* 1 | EU787042 | EU787096 | EU787310 | AY905211 | EU787253 | EU787279 | EU787386 | EU787231 |
| HBS 108703 | *Emys blandingii* 2 | EU787037 | EU787094 | EU787308 | EU787176 | EU787254 | EU787280 | EU787387 | EU787232 |
| HBS 108702 | *Emys blandingii* 3 | EU787040 | EU787100 | EU787314 | EU787181 | EU787255 | EU787281 | EU787388 | EU787233 |
| HBS 39753 | *Emys marmorata* 2 | EU787053 | EU787110 | EU787324 | AY905237 | EU787257 | EU787283 | EU787390 | EU787235 |
| HBS 39806 | *Emys marmorata* 1 | EU787044 | EU787101 | EU787315 | AY905217 | EU787256 | EU787282 | EU787389 | EU787234 |
| HBS 39814 | *Emys marmorata* 3 | EU787061 | EU787118 | EU787332 | AY905253 | EU787258 | EU787284 | EU787391 | EU787236 |
| HBS 39843 | *Emys marmorata* 4 | EU787051 | EU787108 | EU787322 | AY905235 | EU787259 | EU787285 | EU787392 | EU787237 |
| IPMB 4529 | *Emys orbicularis* 1 | EOR131412 | EU787124 | EU787338 | EU787184 | EU787260 | EU787286 | EU787393 | EU787238 |
| IPMB 4597 | *Emys orbicularis* 2 | EU787065 | EU787125 | EU787339 | EU787185 | EU787261 | EU787287 | EU787394 | EU787239 |
| HBS 108694 | *Emys orbicularis* 3 | EU787071 | EU787152 | EU787366 | EU787212 | EU787262 | EU787288 | EU787395 | EU787240 |
| HBS 108690 | *Emys orbicularis* 4 | EU787073 | EU787154 | EU787368 | EU787214 | EU787263 | EU787289 | EU787396 | EU787241 |
| HBS 108714 | *Glyptemys insclpta* 1 | EU787027 | EU787084 | EU787298 | EU787166 | EU787248 | EU787274 | EU787381 | EU787226 |
| HBS 108715 | *Glyptemys muhlenbergii* 1 | EU787028 | EU787085 | EU787299 | EU787167 | EU787249 | EU787275 | EU787382 | EU787227 |
| HBS 23204 | *Graptemys pseudogeographica* 2 | EU787025 | EU787082 | EU787296 | EU787164 | EU787246 | EU787272 | EU787379 | EU787224 |
| HBS 27240 | *Terrapene c. carolina* 1 | EU787029 | EU787086 | EU787300 | EU787168 | EU787250 | EU787276 | EU787383 | EU787228 |
| HBS 108676 | *Terrapene coahuila* 1 | EU787030 | EU787087 | EU787301 | EU787169 | EU787251 | EU787277 | EU787384 | EU787229 |
| HBS 27363 | *Terrapene ornata* 1 | EU787031 | EU787088 | EU787302 | EU787170 | EU787252 | EU787278 | EU787385 | EU787230 |
| HBS 23001 | *Trachemys s. elegans* 1 | EU787024 | EU787081 | EU787295 | EU787163 | EU787245 | EU787271 | EU787378 | EU787223 |
| HBS 109887 | *Chelonia mydas* | EU787021 | EU787076 | EU787292 | EU787159 | AY687907 | EU787266 | EU787373 | EU787219 |
| HBS 16255 | *Platysternon megacephalum* | NC_007970  MVZ230486 | EU787077 | EU787293 | EU787160 | AY687905 | EU787267 | EU787374 | NA |
| HBS 109888 | *Psammobates pardalis* | NC_007694  MVZ241333 | EU787078 | NA | EU787162 | AY687912 | EU787269 | EU787376 | EU787221 |
| HBS 109889 | *Heosemys spinosa* | EU787022 | EU787079 | EU787294 | EU787161 | AY687913 | EU787268 | EU787375 | EU787220 |
| MVZ 230476 | *Mauremys mutica* 1 | FJ770585 | EF011276 | EF011232 | EF011426 | FJ770713 | FJ770803 | FJ770849 | FJ770895 |
| MVZ 230487 | *Mauremys mutica* 3 | EU787023 | EU787080 | EF011233 | EF011427 | EU787244 | EU787270 | EU787377 | EU787222 |

FMNH = Field Museum of Natural History, HBS = tissue collection of H. Bradley Shaffer, IPMB = Institute for Pharmacy and Molecular Biotechnology, Heidelberg, Germany, MVZ = Museum of Vertebrate Zoology, Berkeley, California. NA = missing data.
